# MicroRNA-103 Protects Coronary Artery Endothelial Cells against H_2_O_2_-Induced Oxidative Stress via BNIP3-Mediated End-Stage Autophagy and Antipyroptosis Pathways

**DOI:** 10.1155/2020/8351342

**Published:** 2020-02-21

**Authors:** Yiran Wang, Xianjing Song, Zhibo Li, Ning Liu, Youyou Yan, Tianyi Li, Wei Sun, Yinuo Guan, Ming Li, Yibo Yang, Xingru Yang, Bin Liu

**Affiliations:** Department of Cardiology, The Second Hospital of Jilin University, Changchun, Jilin, China

## Abstract

Endothelial cell damage caused by oxidative stress is widely considered to be a triggering event in atherosclerosis (AS). However, the specific effect elicited by autophagy in endothelial cells undergoing oxidative stress remains controversial, especially during end-stage autophagy. The inhibition of end-stage autophagy has been reported to increase cell pyroptosis and contribute to endothelial damage. Several studies have shown that microRNA-103 is involved in end-stage autophagy; however, its specific mechanism of action is not yet characterized. In this study, we addressed the regulatory role of miR-103 in autophagy during oxidative stress of endothelial cells. Hydrogen peroxide (H_2_O_2_) treatment was used as an *in vitro* model of oxidative stress. MTS and ROS levels were measured to evaluate cell activity. qRT-PCR was used to detect the expression of miR-103. Autophagy was examined using western blot, immunofluorescence staining, and electron microscopy, while western blot analysis detected pyroptosis-related proteins. Results show that miR-103 expression decreased under oxidative stress. Further, miR-103 repressed transcription of Bcl-2/adenovirus E1B 19 kDa interacting protein (BNIP3). The oxidative stress caused by H_2_O_2_ caused cell damage from 2 hours (*P* < 0.05) and increased the level of intracellular reactive oxygen species (*P* < 0.05); at the same time, the damage could be further aggravated by the stimulation of bafA1 (*P* < 0.05). Under the stimulation of H_2_O_2_, the expression of miR-103 decreased (*P* < 0.05). However, high expression of miR-103 could reduce the accumulation of LC3II and P62 (*P* < 0.05) by inhibiting the downstream target gene Bcl-2/adenovirus E1B 19 kDa interacting protein (BNIP3), thus reducing the occurrence of cell pyroptosis (*P* < 0.05). This process could be blocked by end-stage autophagy inhibitor bafA1 (*P* < 0.05), which further indicated that miR-103 affected cell injury by autophagy. On the contrary, the low expression of miR-103 promoted the accumulation of autophagy protein and increased the occurrence of pyroptosis (*P* < 0.05). In conclusion, inhibition of miR-103 restrained end-stage of autophagy by regulating BNIP3, thus changing the occurrence of cell pyroptosis.

## 1. Introduction

Atherosclerosis (AS) is a chronic inflammatory response accompanied by a myriad of serious complications, representing an important cause of mortality and disability worldwide [1, 2]. Oxidative stress is widely considered to be an initiator of AS, resulting in the production of ROS, which in turn inactivates or reduces the expression of antioxidant proteins; oxidizes nucleic acids, lipids, and proteins; and ultimately leads to the destruction of cell structure and function [3]. Moreover, oxidative stress activates inflammatory factors such as nuclear factor *κ*B (NF-*κ*B) and adaptor protein complex-1 (AP-1), which stimulate the expression of vascular adhesion molecules, inducing migration of immune cells to the damaged vascular endothelium [4]. In addition, many studies have found that autophagy affects oxidative stress-induced injury of endothelial cells in cardiovascular diseases. However, the specific role of autophagy in cell response to oxidative stress in AS-related vascular injury has not yet been characterized.

Autophagy is a physiological cellular process that permits lysosomal catabolism of damaged organelles and errant proteins. The process is divided into early autophagy and end-stage autophagy, relying on the formation of autolysosome, both of which have different mechanisms by which they respond to cell injury [5, 6]. Although the role of autophagy in oxidative stress has been widely examined in recent years, few studies have specifically addressed the role of end-stage autophagy, and those that have, yielded controversial results [7, 8]. For instance, ROS were found to inhibit the clearance of autophagic lysosomes at the end of the ischemia-reperfusion period, thus promoting the occurrence of cell apoptosis and aggravating cell damage [9], while other studies have suggested that promoting autophagy can reduce the inflammatory response caused by mitochondrial damage [10]. Recently, a new type of programmed cell death, pyroptosis has been described, which consists of focal cell death primarily occurring through recognition of pathogen-associated molecular patterns (PAMPs) and danger-associated molecular patterns (DAMPs), which activates the immune cell receptor and promotes the expression of NOD-like receptor family, pyrin domain containing 3 (NLRP3), caspase 1, and interleukin- (IL-) 1*β*, as well as other inflammatory factors [11, 12]. The NLRP3 inflammasome, specifically, is delivered to autolysosomes by autophagy biomarkers LC3 and P62 [13]. Additionally, miRs play an important role in regulating autophagy in many diseases, especially cardiovascular diseases [14–16]; however, further studies are needed to investigate the role of miRs in the response to endothelial cell inflammatory injury through end-stage autophagy.

MicroRNAs, composed of 20-40 nucleotides, are highly conserved single-stranded noncoding RNA, that regulate gene expression in many biological processes [17, 18]. The gene coding region for miR-103 is located on human chromosome 5 and belongs to the miR-103/107 gene family [19], which is widely expressed in various tissues. In fact, several studies have shown that inhibiting the expression of miR-103 may result in a protective effect in cardiovascular diseases. Therefore, during hyperlipidemia, miR-103-mediated inhibition of lncwdr59 promotes endothelial maladaptation by impairing EC regeneration and increasing mitotic abnormalities. Alternatively, reduced expression of miR-103 limits the occurrence of AS by preventing miR-103-induced lncwdr59 inhibition, thereby protecting endothelial cells from endothelial damage and reducing the targeted inhibition of Kruppel-like factor 4 (kfl4) [20]. Other studies have found that low miR-103 expression results in enhanced expression of the downstream target gene, phosphatase, and tensin homolog deleted on chromosome 10 (PTEN), which inhibits the p38 mitogen-activated protein kinase (MAPK) signaling pathway and reduces the inflammatory response and ER stress response in endothelial cells exposed to oxidized low-density lipoprotein (ox-LDL) [21]. Moreover, miR-103 knockout reduces the necrosis of H9C2 cells treated with H_2_O_2_, by regulating the downstream target gene Fas-associating protein with death domain (FADD) and preventing the formation of the ripk1/ripk3 complex [22]. Conversely, in cardiomyopathy, miR-103 overexpression directly targets transient receptor potential vanilloid 3 (TRPV3), a nonspecific calcium channel; reduces calcium influx; decreases the expression of autophagy markers, LC3II, and beclin-1; increases p62; inhibits autophagy; and reduces cell surface area thereby attenuating stress-induced cardiomyopathy [23]. In addition, in human limbal endothelial cells, Inh-103 was found to inhibit the phosphorylation of dynamin1 by targeting phospholipase D1 (PLD1) and phospholipase D2 (PLD2), thus inhibiting end-stage autophagy [24]. Hence, the role of miR-103 in cardiovascular diseases is far from clear and required further in-depth comprehensive studies to further characterize it.

In this study, we investigated the role of miR-103 in autophagy and oxidative stress response-induced atherosclerosis. Results show that miR-103 was decreased in H_2_O_2_-treated human coronary artery endothelial cells (HCAECs), which resulted in BNIP3 upregulation, and inhibition of the interaction between autophagosomes and lysosomes, resulting in accelerated cell pyroptosis. Alternatively, miR-103 upregulation induced antipodal effects. Finally, we show that inhibiting BNIP3 expression reduces Inh-103-induced injury, thereby alleviating the cell inflammatory reaction. Overall, our results demonstrate that miR-103 protects endothelial cells from oxidative stress by suppressing BNIP3.

## 2. Materials and Methods

### 2.1. Cell Culture and Treatment

Human coronary artery endothelial cells (cell bank of Shanghai Chinese Academy of Sciences) were cultured in DMEM/high-glucose medium (Gibco, USA, Cat No: SH30022.01) supplemented with 10% fetal bovine serum (FBS; HyCLone, USA, Cat No: SH30084.03) and 1% penicillin streptomycin (HyCLone, USA, Cat No: sv30010), at 37°C in 5% CO_2_. The cells were digested and passaged with pancreatin (1 : 250, Solarbio, China, Cat No: 825M042). Cells in the experimental group were treated with 1000 *μ*m H_2_O_2_ for 1 h, 2 h, 4 h, 6 h, or 8 h. To inhibit end-stage autophagy, cells were treated with the autophagy inhibitor bafA1 (Selleck, China, Cat No: S413) at 0.1 *μ*m for 4 h. To inhibit early-stage autophagy, we used the autophagy inhibitor 3MA (Selleck, China, Cat No: S2767) at 0.1 *μ*m for 24 h.

### 2.2. MTS

Cell viability was measured with the CellTiter 96 Aqueous One Solution Cell Proliferation Assay (MTS, Promega, USA, Cat No: G3582) according to the manufacturer's instructions. HCAECs were seeded in 96-well plates (1 × 10^4^ cells/well) for 24 h, after which H_2_O_2_ and autophagy inhibitors were added for further stimulation. After reaching the designated time point, the original culture solution was removed and discarded and the wells were washed with phosphate buffered saline (PBS) (1×, EUROIMMUN, Cat No: F160811CD), followed by addition of 10 *μ*L/well MTS reagent and 90 *μ*L/well DMEM and incubation at 37°C for 1 h in the dark. The absorbance was measured with a Varioskan Flash reader (Varioskan Flash, Thermo Scientific, USA) at a wavelength of 490 nm.

### 2.3. ROS

The intracellular ROS level was measured by DCFH-DA (Bestbell Biotechnology, Shanghai, Cat No: BB-47053). HCAECs (1 × 10^4^ cells/well) in logarithmic growth phase were seeded in 24-well plates and grown to 80% confluence. The cells were then washed twice with PBS, after which DCFH-DA diluted 1 : 1,000 in serum-free DMEM was added and incubated at 37°C in the dark for 20 min. Cells were then washed again with PBS and examined with a binocular-inverted fluorescence microscope (OLYMPUS, Japan).

### 2.4. Lentivirus Transfection

Cells with high miR-103 expression (Pre-103) and low miR-103 expression (Inh-103) were generated by lentivirus transfection. An empty plasmid (miR-NC) was used as a control. HCAECs (5 × 10^4^ cells/well) were cultured with serum-free DMEM, which diluted the lentivirus to 10^7^/mL (mol = 10) (Sangon Biotech, China). After 48 h of transfection, the culture medium and virus were removed and 2 mL of normal culture medium was added for an additional 24 hours.

On the next day, the culture medium was removed according to the resistance of lentivirus vector and 2 mL of culture medium containing 3 *μ*g/mL puromycin aminonucleoside (Abcam, UK, Cat No: ab142726) was added. The cell cultures were allowed to incubate and passaged when necessary. Next, puromycin containing medium culture cells were continuously used for a certain number of generations. Finally, the transfection efficiency was evaluated by fluorescence microscopy, qRT, PCR, and western blot.

The transfection sequences were as follows:

Pre-103: 5′-AGCAGCAUUGUACAGGGCUAUGA-3′;

Inh-103: 5′-UCAUAGCCCUGUACAAUGCUGCU-3′.

### 2.5. RNA Interference

To analyze the role of BNIP3 in cells with low miR-103 expression, BNIP3 expression was suppressed by siBNIP3 oligo (GenePharma, China). Empty plasmids (pGPU6) (GenePharma, China, Cat No: 191030) were used as a control. The 1OD plasmid powder was centrifuged at 10,000 rpm for 1 min, and 125 *μ*L RNase-free water was added. The final RNA concentration was determined to be 20 *μ*m. According to the calculated concentration, the RNA solution was combined with an equal volume of siRNA translate mate plus and allowed to stand for 15 min. After cell transfection for 36-72 h, stably transfected cells were selected by medium containing 0.5 mg/mL G418 (Biotechnology, Shanghai, Cat No: 60220ES03).

Transfection sequence: 5′-GGGCAUAUUCUCUGCAGAAdTdT-3′.

### 2.6. Quantitative Real-Time PCR (qRT-PCR)

Trizol reagent (Ambion, USA, Cat No: 15596-026) was used to extract total RNA from control, treated, and transfected cells. The concentration (ng/*μ*L) of microRNA was quantified and evaluated for purity by a NanoDrop ultraviolet spectrophotometer (Thermo Scientific, USA). RNA was reverse-transcribed into cDNA using an mRNA reverse transcription kit and an miRNA reverse transcription kit (TransGen Biotech, Beijing, China, Cat No: M31121) according to the manufacturers' instructions. Quantitative real-time PCR was performed using the primers listed below. Gene specific primers and SYBR Green Master Mix (TransGen Biotech, Beijing, China, Cat No: N10627) were used to detect the expression of miR-103. U6 was used to standardize miRNA. The results are expressed as multiples of U6 and calculated using the 2^-△△CT^ method.

The primer sequences used were as follows:

U6 (human) F: 5′-CTCGCTTCGGCAGCACA-3′ R: 5′-AACGCTTCACGAATTTGCGT-3′; miR-103 (human) F: 5′-AGAGCAGCATTGTACAGGGCTATGA-3′.

### 2.7. Western Blot

Protease inhibitor (Applygen, China, Cat No: P1265), phosphatase inhibitor (Beyotime, China, Cat No: BB18031), and high-efficiency RIPA protein lysate (Beyotime, China, Cat No:) were used to lyse HCAECs. The cell lysates were separated on a 12% SDS-PAGE gel and transferred to a PVDF membrane (Millipore, Bedford, MA, USA). Antibodies against LC3 (1 : 1000, CST, USA, Cat No: 3868S), p62 (1 : 1000, CST, USA, Cat No: 16177S), mTOR (1 : 1000, CST, USA, Cat No: 2983S), p-mTOR (1 : 1000, CST, USA, Cat No: 5536S), NLRP3 (1 : 1000, Abcam, UK, Cat No: ab214185), caspase1 (1 : 1000, Abcam, UK, Cat No: ab179515), IL-1*β* (1 : 1000, Abcam, UK, Cat No: ab9722), BNIP3 (1 : 1000, Abcam, UK, Cat No: ab219609), horseradish peroxidase- (HRP-) combined secondary antibodies (1 : 2000, Goat Anti-Mouse IgG HRP Affinity Purified PAb, Santa Cruz, USA, Cat No: sc2005; Goat Anti-Rabbit IgG HRP Affinity Purified PAb, Santa Cruz, USA, Cat No: sc2357), goat anti-mouse IgG HRP affinity purified systems, and goat anti-rabbit IgG HRP were used for 50 minutes at room temperature. Also, ECL fluorescent developer (Thermo Scientific, USA, Cat No: 17295) and LAS3000 Imager (Fuji Photo Film Co, Ltd.) were used to detect protein bands. Image J software was used to calculate the intensity of the protein bands. PVDF membranes were washed with membrane stripping buffer 1 (GenStar, China, Cat No: ISEQ00010) for 1 h and then reincubated with antibodies overnight. SPSS 20.0 statistical software was used for analysis. Actin (1 : 1000, Bioss Technology, China, Cat No: P60709) was used as an internal reference.

### 2.8. IF

HCAECs were fixed with 4% paraformaldehyde (Biosharp Biotechnology, China, Cat No: BL539A) for 30 min and permeabilized with 0.1% Triton X-100 (Biosharp, China, Cat No: 1805132) for 20 min. After washing with PBS, 5% bovine serum albumin (BSA) in PBS was added and incubated at room temperature for 2 h. Cells were then incubated with anti-LC3 (1 : 500, CST, USA, Cat No: 3868S) and p62 (1 : 200, CST, USA, Cat No: 88588S) antibodies overnight. The second fluorescence was incubated in the dark for 1 h. The nuclei were stained with DAPI for 5 min, and images were evaluated by confocal microscopy (OLYMPUS, Japan).

### 2.9. Transmission Electron Microscopy (TEM)

HCAECs were stored overnight in 2.5% dehydrated (Coolaber, China, Cat No: SL29143550) with ethanol and embedded in EPON resin. Ultrathin sectioning was performed in representative areas and observed at 80 kV accelerating voltage using a HITACHI TEM system. The cell ultrastructure was evaluated using an AMT imaging system (Advanced Microscopy Techniques Co, USA).

### 2.10. Statistical Analysis

All experiments were repeated three times independently, and results were expressed as mean ± standard deviation (SD). The statistical analysis was carried out with GraphPad 6.0 statistical software (GraphPad Software, San Diego, CA, USA). *P* values were calculated by ANOVA. *P* < 0.05 was considered as statistically significant.

## 3. Result

### 3.1. Oxidative Stress Induces HCAEC Injury

**Figure 1:**
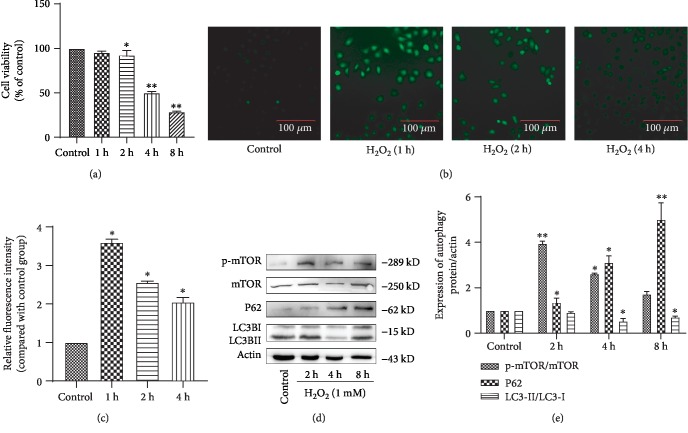
Oxidative stress induces HCAEC injury. (a) Cell viability was studied after treated with H_2_O_2_ (0 h, 1 h, 2 h, 4 h, and 8 h) by MTS assay. (b, c) ROS analysis was performed for intracellular ROS level. (d, e) Relative expression of autophagy proteins was detected by western blot. (e) Data are shown as mean ± SD. ^∗^*P* < 0.05, ^∗∗^*P* < 0.01 (^s^compared with the control group).

**Figure 2:**
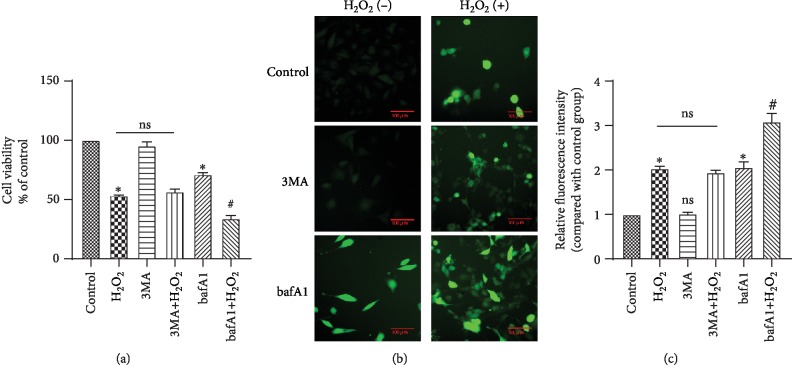
Effect of inhibition of end-stage autophagy and early-stage autophagy on HCAECs. (a) Cell viability was studied by MTS assay. (b, c) ROS analysis was performed for intracellular ROS level. Data are shown as mean ± SD. ^∗^*P* < 0.05, ^∗∗^*P* < 0.01, ^#^*P* < 0.05 (^∗^compared with the control group, ^#^compared with the bafA1 group).

*In vitro* oxidative stress response was stimulated by cell treatment with H_2_O_2_. In accordance with previous studies, the degree of H_2_O_2_-induced cell damage was time- and concentration-dependent [19]. The damage induced by 1 mM H_2_O_2_ in cells treated for different times was evaluated with an MTS kit (Figure 1(a), ^∗^*P* < 0.05, ^∗∗^*P* < 0.01). Cell damage became apparent after 2 h of treatment, with 50% cell death noted at approximately 4 h. In addition, H_2_O_2_ stimulation significantly increased intracellular ROS, which contributed to the induction of inflammatory cell damage^3^ (Figures 1(b) and 1(c), ^∗^*P* < 0.05). Moreover, when the cells were stimulated with H_2_O_2_ (1 mM) for 2 h, 4 h, and 8 h, acute cell damage caused by H_2_O_2_ significantly increased the p-mTOR/mTOR ratio and the level of p62, whereas the LC3II/LC3I ratio decreased, indicating that autophagy was inhibited (Figures 1(d) and 1(e),^∗^*P* < 0.05, ^∗∗^*P* < 0.01). To further demonstrate that H_2_O_2_ inhibited autophagy, we examined the formation of autophagosomes and autolysosomes by immunofluorescence staining for LC3 and p62 and by transmission electron microscopy. Results clearly show that autophagy was reduced (Figures 2(c) and 2(d)), which agreed with the western blot results. We, therefore, choose 4 h as the most appropriate time for the follow-up experiment. Cumulatively, these results indicate that H_2_O_2_ induced inflammatory cell death and inhibited autophagy in HCAECs.

### 3.2. Inhibition of End-Stage Autophagy Aggravates HCAEC Injury Caused by Oxidative Stress

To further examine the role of autophagy in oxidative stress, cells were treated with 3-mA, an inhibitor of early-stage autophagy, and with bafa1, an inhibitor of end-stage autophagy, prior to H_2_O_2_ stimulation. Bafa1 is a proton pump inhibitor, commonly used to inhibit the binding of autophagosomes to lysosomes [6]. The experimental groups were designed as follows: control group, H_2_O_2_ stimulation group, 3MA stimulation group, 3MA+H_2_O_2_ stimulation group, bafA1 stimulation group, and bafA1+H_2_O_2_ stimulation group. The cell viability assays showed that bafA1 significantly reduced cell survival and increased cell damage, while 3MA had no effect on cell survival. In addition, the cell survival rate of the bafA1+H_2_O_2_ group was further reduced compared to the H_2_O_2_ group, and the injury caused by H_2_O_2_ was aggravated, while no significant changes were observed in the 3MA+H_2_O_2_ group, compared to the H_2_O_2_ group (Figure 2(a), ^∗^*P* < 0.05, ^#^*P* < 0.05). In addition, the green fluorescence intensity detected by the ROS kit showed that 3MA had no effect on the production of active oxygen. Alternatively, bafA1 significantly increased the ROS green fluorescence intensity, indicating that it promoted the production of active oxygen (Figures 2(b) and 2(c), ^∗^*P* < 0.05, ^#^*P* < 0.05). Notably, 3MA did not play a significant role in acute oxidative stress injury, while bafA1 increased cell damage. However, other studies reported that 3MA reversed the protective effect of autophagy on cells in oxidative stress response by inhibiting autophagy [25]. Generally, autophagy is observed following exposure of cells to stimulants (such as high glucose) or exogenous oxidants for an extended period of time, usually 24–48 h. Eventually, however, the initial oxidants become consumed by the system, after which their effects have not been well studied [26].

**Figure 3:**
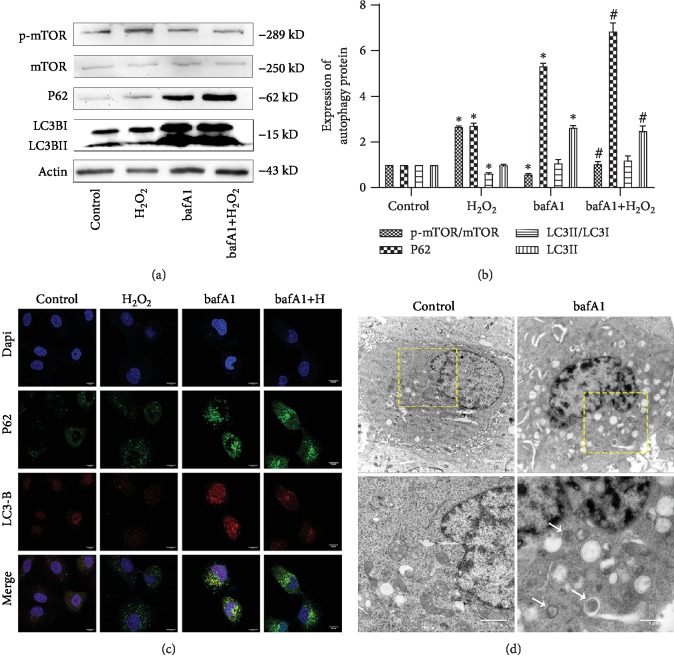
Inhibition of end-stage autophagy aggravates HCAEC injury. (a, b) Relative expression of autophagy proteins was detected by western blot. Data are shown as mean ± SD, ^∗^*P* < 0.05, ^#^*P* < 0.05, ^∗^compared with the control group, ^#^compared with the bafA1 group. (c) Double staining of HCAECs with autophagy marker LC3B (red) and P62 (green). (d) Representative TEM images of HCAECs, HCAECs 24 h treatment with bafA1. Representative high-magnification TEM images show autophagosomes and autolysosomes (arrow). Bars, 2 *μ*m; bars, 1 *μ*m.

Next, we focused exclusively on end-stage autophagy by examining the LC3II/actin ratio which is considered to represent the autophagy level of end-stage cells [27]. Western blot analysis showed that following bafA1 treatment, no significant change in the LC3II/LC3I ratio occurred, whereas the LC3II/actin ratio was significantly higher than in the control group. Further, LC3II and LC3I increased synchronously after treatment with bafA1. Similarly, although there was no difference in the LC3II/LC3I ratio between bafA1+H_2_O_2_ cells and H_2_O_2_-treated cells group, the LC3II level was significantly higher in the H_2_O_2_-treated group. Moreover, BafA1 increased the p62/actin ratio and decreased the p-mTOR/mTOR ratio (Figures 3(a) and 3(b), ^∗^*P* < 0.05, ^#^*P* < 0.05). In addition, immunofluorescence staining showed that LC3 and p62 were colocalized after treatment with the proton pump inhibitor (Figure 3(c)). Large numbers of vacuoles and double-membrane autophagic vesicles were also observed via TEM in Bafa1-treated cells (Figure 3(d)). These results suggest that inhibition of end-stage autophagy increased the accumulation of p62 and LC3II in cells, thereby aggravating cell injury.

### 3.3. Oxidative Stress Downregulates miR-103 and High Expression of miR-103 Affects End-Stage Autophagy

**Figure 4:**
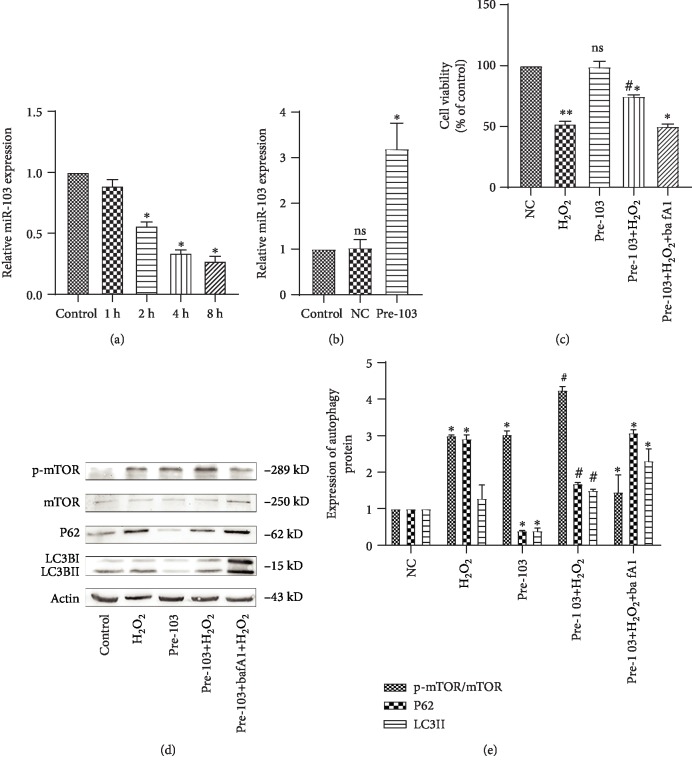
MiR-103 upregulation alleviates oxidative stress in HCAECs. (a) Relative expression of miR-103 after treated with H_2_O_2_ was detected by qRT-PCR, ^∗^*P* < 0.05, ^∗^compared with the NC group. (b) Relative expression of miR-103 was detected by qRT-PCR after treatment with NC or pre-103, ^∗^*P* < 0.05, ^∗^compared with the control group. (c) Cell viability was studied by MTS assay, ^∗∗^*P* < 0.01, ^#^*P* < 0.05, ^△^*P* < 0.05, ^*δ*^*P* < 0.05, ^∗^compared with the NC group, ^#^compared with the H_2_O_2_ group, ^△^compared with the pre-103 group, ^*δ*^compared with the pre-103+H_2_O_2_ group. (d, e) Relative expression of autophagy proteins was detected by western blot. Data are shown as mean ± SD, ^∗^*P* < 0.05, ^△^*P* < 0.05, ^#^*P* < 0.05, ^∗^compared with the NC group, ^#^compared with the H_2_O_2_ group, ^△^compared with the pre-103+H_2_O_2_ group.

We next used qRT-PCR to examine the effect of 1 mM H_2_O_2_ stimulation on miR-103 expression at different time points (1, 2, 4, and 8 h). After 2 h, H_2_O_2_ significantly inhibited the expression of miR-103 compared to control cells (Figure 4(a), ^∗^*P* < 0.05, ^∗∗^*P* < 0.01, ^#^*P* < 0.05, ^△^*P* < 0.05). This result was consistent with similar studies by Xu [19]. In particular, H_2_O_2_ inhibited miR-103 expression in HUVECs. Following lentivirus-mediated miR-103 transfection, miR-103 levels increased by approximately three times, as determined by qRT-PCR, indicating that the transfection was successful (Figure 4(b), ^∗^*P* < 0.05).

Additionally, the MTS results revealed that, compared to the NC+H_2_O_2_ group, treatment with pre-103+H_2_O_2_ increased the survival rate from 50% to approximately 70%. However, the protective effect of pre-103 was weakened by bafA1, indicating that pre-103 affected cell activity through end-stage autophagy (Figure 4(c), ^∗∗^*P* < 0.01, ^#^*P* < 0.05, ^△^*P* < 0.05, ^*δ*^*P* < 0.05). Further, western blot results showed that pre-103 decreased LC3II and p62 levels and increased the p-mTOR/mTOR ratio (Figures 4(d) and 4(e), ^∗^*P* < 0.05, ^△^*P* < 0.05, ^*δ*^*P* < 0.05). However, LC3 accumulation was not detected by immunofluorescence staining.

**Figure 5:**
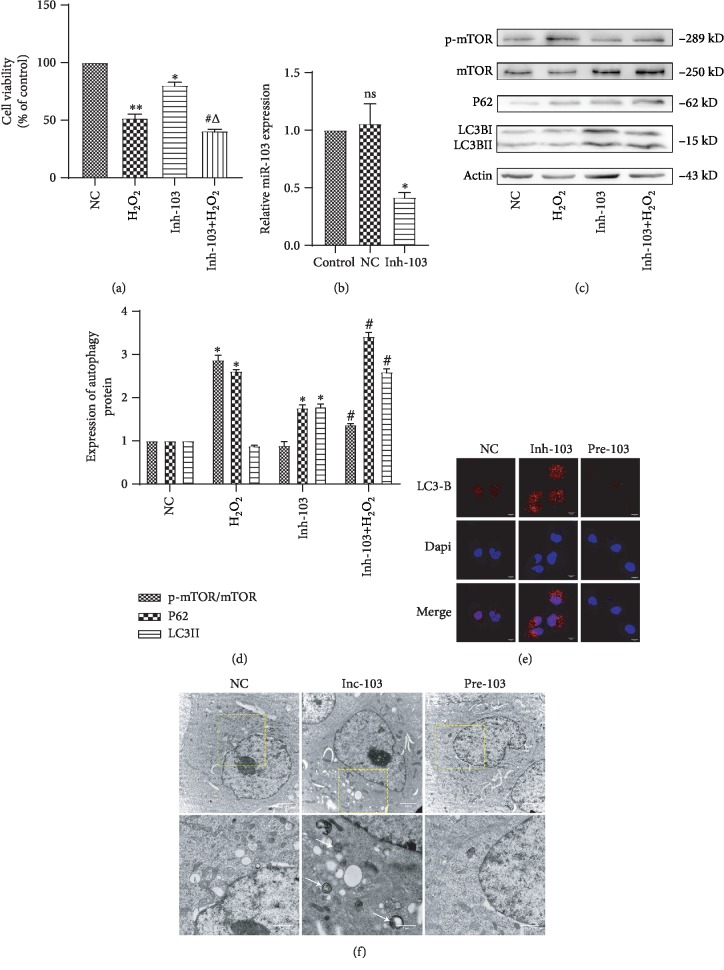
Downregulating miR-103 aggravates injury by inhibiting end-stage autophagy in HCAECs. (a) Cell viability was studied by MTS assay, ^∗∗^*P* < 0.01, ^∗^*P* < 0.05, ^#^*P* < 0.05, ^△^*P* < 0.05, ^∗^compared with the NC group, ^#^compared with the H_2_O_2_ group, ^△^compared with the pre-103 group. (b) Relative expression of miR-103 was detected by qRT-PCR after treatment with NC or Inh-103, ^∗^*P* < 0.05, ^∗^compared with the control group. (c, d) Relative expression of autophagy proteins was detected by western blot. Data are shown as mean ± SD, ^∗^*P* < 0.05, ^#^*P* < 0.05, ^∗^compared with the NC group, ^#^compared with the H_2_O_2_ group. (e) Staining of NC, Inh-103, and pre-103 with autophagy marker LC3B (red), bar, 10 *μ*m. (f) Representative TEM images of NC, Inh-103, pre-103. Representative high-magnification TEM images show autophagosomes and autolysosomes (arrow). Bars, 2 *μ*m; bars, 1 *μ*m.

Moreover, compared to the Inh-103 group, few vacuoles or autophagy structures were observed in control-NC cells, as assessed by TEM (Figures 5(e) and 5(f)). In addition, we added bafa1, an end-stage autophagy inhibitor, after H_2_O_2_ treated in pre-103 HCAECs, and showed via western blot analysis that bafA1 reversed the function of high expression miR-103 on inflammatory response and autophagy, and counteracted the protective effects exerted by Pre-103 (Figures 4(d) and 4(e), ^∗^*P* < 0.05, ^△^*P* < 0.05, ^*δ*^*P* < 0.05). Together, these results suggest that pre-103 reduced the accumulation of autophagic ubiquitin-like P62 and LC3II proteins and played an important role in cell protection.

### 3.4. Downregulating miR-103 Aggravates Oxidative Stress-Induced Injury by Inhibiting End-Stage Autophagy in HCAECs

To examine the role of miR-103 in H_2_O_2_-mediated HCAEC injury, the expression of miR-103 was inhibited by lentivirus transfection. Under these conditions, miR-103 level was decreased by approximately 0.45 times, as determined by qRT-PCR, indicating successful transfection (Figure 5(b), ^∗^*P* < 0.05, ^∗∗^*P* < 0.01, ^#^*P* < 0.05, ^△^*P* < 0.05). After transfection with Inh-103, MTS results revealed a significantly reduced cell survival rate (Figure 5(a), ^∗^*P* < 0.05, ^#^*P* < 0.05, ^△^*P* < 0.05). In addition, we found that Inh-103 increased LC3II and p62 accumulation in cells, while Inh-103+H_2_O_2_ significantly reduced p-mTOR/mTOR compared to NC+H_2_O_2_ cells, indicating that miR-103 itself had no effect on the mTOR pathway, while in the presence of H_2_O_2_, it reduced p-mTOR expression, thus inhibiting end-stage autophagy (Figures 5(c) and 5(d), ^∗^*P* < 0.05, ^#^*P* < 0.05). Further, immunofluorescence staining showed a large number of red fluorescence spots in Inh-103-positive cells compared to control cells, suggesting that the autophagic marker protein, LC3B, accumulated in the cytoplasm (Figure 5(e)). Large numbers of vacuoles were also observed in the cells by electron microscopy, and a cargo-containing double-membrane autophagic structure was observed by TEM (Figure 5(f)). Therefore, low miR-103 expression increased accumulation of autophagic molecules in the cells and aggravated cell damage.

### 3.5. Cell Injury Induced by miR-103 Downregulation Is Prevented by Repression of Its Target Gene Bcl2/Adenovirus EIB 19 kDa Interacting Protein 3 (BNIP3)

**Figure 6:**
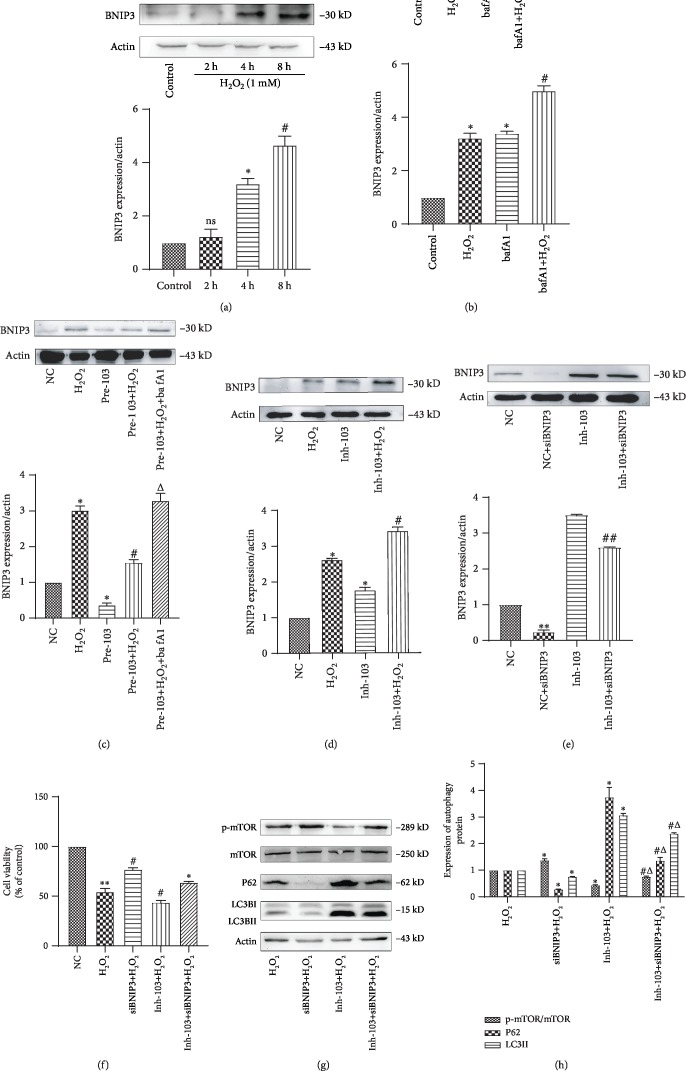
Repression of BNIP3 prevents cell injury induced by miR-103 downregulation. (a) Relative expression of BNIP3 was detected by western blot after treatment with H_2_O_2_. Data are shown as mean ± SD, ^∗^*P* < 0.05, ^∗^compared with the control group. (b) Relative expression of BNIP3 was detected by western blot after treatment with 24 h for bafA1. Data are shown as mean ± SD, ^∗^*P* < 0.05, ^∗^*P* < 0.05, ^#^*P* < 0.05, ^∗^compared with the control group, ^#^compared with the H_2_O_2_ group. (c) Relative expression of BNIP3 was detected by western blot after treatment with pre-103. Data are shown as mean ± SD, ^∗^*P* < 0.05, ^#^*P* < 0.05, ^△^*P* < 0.05, ^∗^compared with the NC group, ^#^compared with the H_2_O_2_ group, ^△^compared with the pre-103+H_2_O_2_ group. (d) Relative expression of BNIP3 was detected by western blot after treatment with Inh-103. Data are shown as mean ± SD, ^∗^*P* < 0.05, ^#^*P* < 0.05, ^∗^compared with the NC group, ^#^compared with the H_2_O_2_ group. (e) Relative expression of BNIP3 was detected by western blot after treatment with, NC, siBNIP3, Inh-103, Inh-103+siBNIP3. Data are shown as mean ± SD, ^∗∗^*P* < 0.01, ^##^*P* < 0.01, ^∗^compared with the NC group, ^#^compared with the Inh-103 group. (f) Cell viability was studied by MTS assay, ^∗∗^*P* < 0.01, ^∗^*P* < 0.05, ^#^*P* < 0.05, ^△^*P* < 0.05, ^∗^compared with the NC group, ^#^compared with the H_2_O_2_ group, ^△^compared with the Inh-103+H_2_O_2_ group. (g, h) Relative expression of autophagy proteins was detected by western blot. Data are shown as mean ± SD, ^∗^*P* < 0.05, ^#^*P* < 0.05, ^△^*P* < 0.05, ^∗^compared with the H_2_O_2_ group, ^#^compared with the SiBNIP3+H_2_O_2_ group, ^△^compared with the Inh-103+H_2_O_2_ group.

Under H_2_O_2_ stimulation, BNIP3 expression increased significantly at 4 h (Figure 6(a), ^∗^*P* < 0.05). Following addition of bafa1, BNIP3 was further increased compared with the control group (Figure 6(b), ^∗^*P* < 0.05, ^#^*P* < 0.05). miR-103 suppression also significantly increased the expression level of BNIP3 compared to the negative control group (Figure 6(d), ^∗^*P* < 0.05, ^#^*P* < 0.05, ^△^*P* < 0.05), while high miR-103 expression downregulated BNIP3, further confirming that BNIP3 is a downstream target of miR-103 (Figure 6(c), ^∗^*P* < 0.05, ^#^*P* < 0.05). In HUVECs as well as other cell types, studies have shown that a binding site for miR-103 exists in the 3′-UTR of BNIP3 [19]. Moreover, by using TargetScan (http://www.targetscan.org/) and miRbase (http://www.microrna.org/microrna/home.do) online tools, we predicted that BNIP3 was a potential target of miR-103.

Next, we explored whether miR-103 could affect autophagy by regulating BNIP3. To this end, sibnip3 plasmids were used to transfect NC and inh-103 cells. Western blot results showed that BNIP3 was successfully suppressed (Figure 6(e), ^∗∗^*P* < 0.01, ^##^*P* < 0.01). MTS results showed that the cell survival rate of siBNIP3+H_2_O_2_ cells was significantly higher compared to that of NC+H_2_O_2_ cells. Moreover, cell activity was significantly higher in inh-103+siBNIP3+H_2_O_2_ cells than in inh-103+H_2_O_2_ cells (Figure 6(f), ^∗∗^*P* < 0.01, ^#^*P* < 0.05, ^△^*P* < 0.05). Finally, western blot analysis revealed that, following siBNIP3 transfection, the LC3II/actin ratio and the level of p62 expression were lower compared to cells with low miR-103 expression (Figures 6(g) and 6(h), ^∗^*P* < 0.05, ^#^*P* < 0.05, ^△^*P* < 0.05). In conclusion, siBNIP3 reversed miR-103-induced damage by preventing inhibition of end-stage autophagy.

### 3.6. Inh-103 Suppresses Autophagy of End-Stage Cells by Regulating BNIP3, Thus Aggravating Cell Pyroptosis

**Figure 7:**
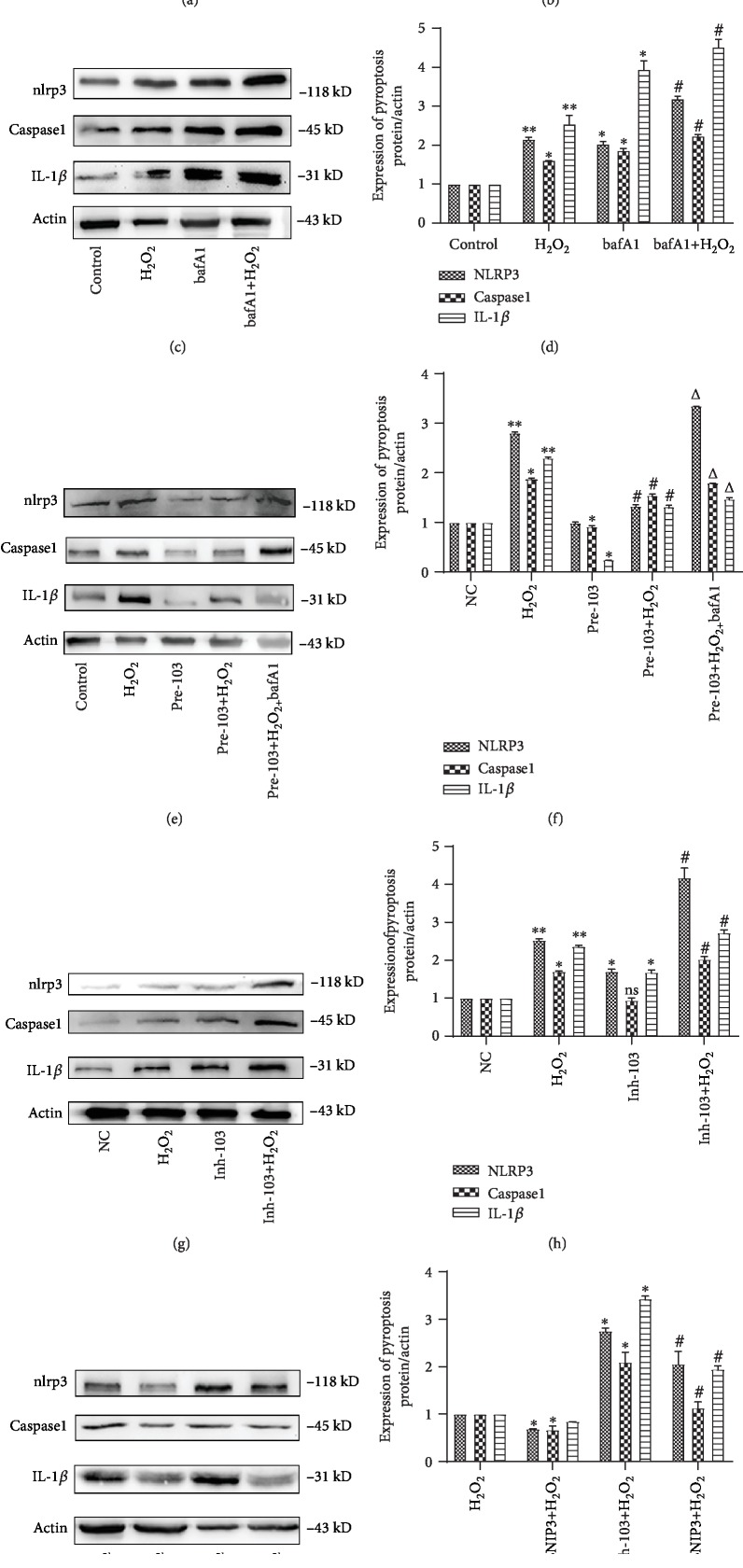
The relationship between miR-103 and pyroptosis in H_2_O_2_-induced HCAECs. (a, b) Relative expression of cell pyroptosis was detected by western blot after treatment with H_2_O_2_. Data are shown as mean ± SD, ^∗^*P* < 0.05, ^∗∗^*P* < 0.01, ^∗^compared with the control group. (c, d) Relative expression of cell pyroptosis was detected by western blot after treatment with 24 h for bafA1. Data are shown as mean ± SD, ^∗^*P* < 0.05, ^∗∗^*P* < 0.01, ^∗^*P* < 0.05, ^#^*P* < 0.05, ^∗^compared with the control group, ^#^compared with the H_2_O_2_ group. (e, f) Relative expression of cell pyroptosis was detected by western blot after treatment with pre-103. Data are shown as mean ± SD, ^∗^*P* < 0.05, ^∗∗^*P* < 0.01, ^#^*P* < 0.05, ^△^*P* < 0.05, ^∗^compared with the NC group, ^#^compared with the H_2_O_2_ group, ^△^compared with the pre-103+H_2_O_2_ group. (g, h) Relative expression of cell pyroptosis was detected by western blot after treatment with Inh-103. Data are shown as mean ± SD, ^∗^*P* < 0.05, ^∗∗^*P* < 0.01, ^#^*P* < 0.05, ^∗^compared with the NC group, ^#^compared with the H_2_O_2_ group. (i, j) Relative expression of cell pyroptosis was detected by western blot after treatment with H_2_O_2_, siBNIP3+H_2_O_2_, Inh-103+H_2_O_2_, Inh-103+siBNIP3+H_2_O_2_. Data are shown as mean ± SD, ^∗^*P* < 0.05, ^#^*P* < 0.05, ^∗^compared with the H_2_O_2_ group, ^#^compared with the Inh-103+H_2_O_2_ group.

A large number of reports have indicated that following inhibition of end-stage autophagy, NLRP3 inflammasome degradation was decreased by accumulated autophagic markers, LC3II and p62. Further, we show that after 2 h of stimulation with 1 mM H_2_O_2_, cell pyroptosis begins to increase and is significantly increased after 4 h, indicating that oxidative stress can cause pyrolytic cell death (Figures 7(a) and 7(b)), ^∗^*P* < 0.05, ^∗∗^*P* < 0.01).

Additionally, IL-1*β*, caspase-1, and NLRP3 levels were significantly higher in the bafA1 group compared to the control group, as well as in the bafA1+H_2_O_2_ group compared to the H_2_O_2_ group (Figures 7(c) and 7(d)), ^∗^*P* < 0.05, ^∗∗^*P* < 0.01, ^#^*P* < 0.05). This indicated that the inhibition of end-stage autophagy could aggravate oxidative stress-induced pyroptosis.

When miR-103 was poorly expressed, IL-1*β* and NLRP3 levels were found to be increased compared to control cells, while in the Inh-103+H_2_O_2_ group, pyrolytic protein was significantly higher than that in the control group (Figures 7(g) and 7(h)), ^∗^*P* < 0.05, ^∗∗^*P* < 0.01, ^#^*P* < 0.05). Alternatively, pre-103 significantly reduced the expression of cell pyroptosis protein (Figures 7(e) and 7(f)), ^∗^*P* < 0.05, ^∗∗^*P* < 0.01, ^#^*P* < 0.05, ^△^*P* < 0.05). However, this effect was prevented by treatment with bafa1. We also found that miR-103 affected end-stage autophagy by regulating BNIP3 and that NLRP3 and caspase-1 expression were lower in the sibnip3+H_2_O_2_ group compared to the H_2_O_2_ group. In addition, pyroptosis marker protein was significantly lower in Inh-103+sibnip3+H_2_O_2_ cells than that in Inh-103+H_2_O_2_ cells (Figures 7(i) and 7(j)), ^∗^*P* < 0.05, ^#^*P* < 0.05). Hence, miR-103 affected end-stage autophagy by regulating BNIP3, thus controlling cell pyroptosis.

## 4. Discussion

In this study, we have described a signaling pathway associated with oxidative stress in coronary atherosclerosis. Our findings demonstrate that H_2_O_2_ decreases the expression of miR-103, resulting in the selective upregulation of BNIP3 in human coronary artery endothelial cells and, therefore, inhibits end-stage autophagy, in the overactivation of cell pyroptosis, with consequent endothelium injury. Moreover, decreased cell autophagy and miR-103 expression were found in oxidatively stressed HCAECs and were accompanied by increased pyroptosis. Consistently, we observed that miR-103 overexpression alleviated oxidative stress-induced HCAEC injury. Conversely, miR-103 downregulation exacerbated coronary cell damage through inhibited end-stage autophagy and accumulation of inflammatory mediators. Moreover, we found that miR-103 downregulation selectively elevated the expression of BNIP3 in HCAECs and exacerbated H_2_O_2_-induced endothelial cell injury. Finally, we showed that BNIP3 downregulation protected HCAECs from oxidative stress injury, an initial event in AS. Our results suggest that miR-103 is a promising therapeutic tool for endothelial cell injury and coronary atherosclerosis-related heart disease.

In cardiovascular diseases, autophagy can protect cells by digesting senescent organelles and metabolic waste, while causing apoptosis and necrosis in the presence of excessive damage [28, 29]. Autophagy has dual effects on AS, a chronic inflammatory disease. Although autophagy can prevent early changes associated with AS by inhibiting inflammatory cells and maintaining the stability of vascular wall cells, it can also aggravate the disease in late AS stage [30].

Autophagy begins with the formation of phagocytic vesicles, which evolve into autophagic bodies that phagocytize substances expressing a degradation marker. This process is defined as early autophagy. Next, in late-stage autophagy, mature autophagosomes combine with lysosomes to form autolysosomes and enzymatically degrade their contents (via acid proteases) [31]. As opposed to early autophagy, which has recently been found to exert protective effects, the role of late autophagy in cells is not fully understood, and further research regarding the relationship between late autophagy and pyroptosis is required. The results of studies show that the V-ATPase subunit, ATP6v0d2, promotes the fusion process of autophagosomes and lysosomes, removes ROS produced by damaged mitochondria, inhibits the pyrolytic promoter, the inflammasome adaptor PYD, and CARD domain-containing protein/apoptosis-associated speck-like protein containing a CARD (PyCard/ASC), and reduces inflammatory cell response [29]. Further, Spalinger et al. reported that the protein tyrosine phosphatase non-receptor type 22 (PTPN22) inhibits the detachment of NLRP3 from autophagic bodies through NLRP3 dephosphorylation in inflammatory bodies, promoting NLRP3 degradation, ultimately resulting in a protective effect [32]. The N-terminal pyrin segment (PYD) of activated NLRP3 can serve as a scaffold to collect downstream inflammatory molecules such as caspase-1 and IL-1*β*. Studies have shown that ubiquitin ligase recognized inflammatory substrates (included NLRP3) and modified them with ploy-Ub chains. Under physiological conditions, p62 functions as a receptor protein, connecting poly-Ub chains with the UBA domain, assisting NLRP3 inflammatory corpuscles in autophagic degradation, thus maintaining intracellular homeostasis [33]. However, when the function of autophagic lysosomes become damaged, an excessive level of p62 causes NLRP3 inflammatory bodies to accumulate in the cell, activating the inflammatory response [13]. Oxidative stress increases lysosomal instability and permeability and decreases autophagy lysosomal activity, leading to the aggregation of NLRP3 inflammatory bodies [34]. In the current study, we show that the levels of p62/sqtsm1 and LC3II, as well as the levels of NLRP3, caspase1, and IL-1*β*, increased after treatment with bafA1, thereby confirming that the activation of NLRP3 inflammatory corpuscles may be related to the intracellular accumulation of p62. Additionally, *in vivo* experiments have shown that inhibition of lysosomal function enhances the formation and activation of NLRP3 inflammatory bodies [35]. Alternatively, pyroptosis has also been reported to have a regulatory role in autophagy. NLRP3 is a key factor in the production of functional LC3II during *Trypanosoma cruzi* infection [36]. NLRP3 is knocked out by gene, and the immature form LC3 (LC3 I) accumulates in the cell, thus affecting late-stage autophagy. NLRP3 binds to mTOR and promotes its phosphorylation, resulting in autophagy inhibition and exacerbation of the inflammatory response. Moreover, NLRP3 silencing promotes LC3 II and p62 degradation and reduces mTOR phosphorylation [37, 38]. In cardiovascular diseases, the effect of charring on end-stage autophagy needs to be further investigated.

In recent years, a large number of studies have shown that miR-103 is implicated in cardiovascular diseases [39, 40]. Clinical trials demonstrated that the level of miR-103 in peripheral circulation blood is generally reduced in patients with diabetes or heart failure [36]. In addition, Vacca et al. showed that miR-103 is significantly reduced in the epicardial adipose tissue of patients with coronary heart disease [41]. However, the protective effect of miR-103 downregulation remains controversial. For instance, it was found that in ApoE(-/-) mice administered a high-fat diet that miR-103 downregulation inhibits KLF4, thus reducing endothelium membrane fluidity and monocyte adhesion, as well as the occurrence of AS [42]. In addition, low miR-103 expression reduces the formation of the ripk1/ripk3 complex by activating FADD and effectively reducing the occurrence of myocardial necrosis induced by oxidative stress [22]. Moreover, in hyperlipidemia, miR-103 promotes endothelial cell regeneration, reduces or suppresses PTEN/MAPK signaling by promoting the expression of lncwdr59, and attenuates cell damage caused by endoplasmic reticulum stress [20, 21]. However, other studies suggest that low miR-103 expression may cause cell damage. MiR-103 can alleviate cardiomyocyte hypertrophy by inhibiting TRPV3, reducing autophagy and calcium influx [23]. In addition, miR-103 promotes the proliferation of pulmonary arterial smooth muscle cells (PASMCs) and relieves pulmonary hypertension by regulating the hypoxia-inducible factor-1*β* (HIF-1*β*) pathway [43]. In our study, the expression of miR-103 was found to be decreased by H_2_O_2_-induced acute oxidative stress in a time and dose-dependent manner. miR-103 downregulation promoted the expression of LC3II and p62 and increased the occurrence of pyroptosis. Immunofluorescence showed that in cells with low miR-103 expression, LC3 showed obvious aggregated fluorescent spot, indicating that autophagy was activated or that autolysosome decomposition was inhibited. However, the high expression of miR-103 caused decreased LC3II and p62 expression in cells. Other studies found that low miR-103 expression promotes the phosphorylation and deactivation of dynamin-1, thus reducing the activity of lysosomes, as well as self-elimination of senescent organelles and misfolded proteins [27]. In addition, low miR-103 expression promotes the expression of inflammatory factors. We, therefore, speculate that the low expression of miR-103 reduces NLRP3 clearance by inhibiting end-stage autophagy.

Furthermore, we show that BNIP3 was negatively regulated by miR-103. BNIP3 suppression reduced the inhibition of autolysosomal function and the subsequent inflammatory response caused by low expression of miR-103. The study of Xu showed that BNIP3 downregulation reduced the level of ROS produced by mitochondrial damage [19]. In addition, BNIP3 was reported to bind to cytoplasmic trap snap29, competitively inhibiting the interaction between snap29 and the lysosomal trap, vamp8, and blocking the binding of autophagy bodies to lysosomes [44]. Consistent with our results, a study focusing on myocardial ischemia reperfusion, found that high BNIP3 levels cause p62 accumulation. In addition, the interaction between the transmembrane domain of BNIP3 and LC3II promotes the clearance of damaged mitochondria [45].

In conclusion, our results demonstrate that miR-103 suppression accelerates H_2_O_2_-induced cell pyroptosis and end-stage autophagy through BNIP3 upregulation in HCAECs. Our study may serve to clarify the role of end-stage autophagy and oxidative stress in cardiovascular diseases.

## Data Availability

The data used to support the findings of this study are included within the article.
